# Adaptive metabolic pattern biomarker for disease monitoring and staging of lung cancer with liquid biopsy

**DOI:** 10.1038/s41698-018-0059-9

**Published:** 2018-08-08

**Authors:** Manuel Garcia-Algar, Ana Fernandez-Carrascal, Ana Olano-Daza, Luca Guerrini, Neus Feliu, Wolfgang J. Parak, Roger Guimera, Eduardo Garcia-Rico, Ramon A. Alvarez-Puebla

**Affiliations:** 1 Department of Physical Chemistry, Universitat Rovira i Virgili, Marcel•lí Domingo 1, 43007 Tarragona, Spain; 2 Department of Medical Oncology, Hospital Universitario HM Torrelodones, Castillo de Olivares s/n, 28250 Torrelodones Madrid, Spain; 3 Karolinska Institutet, Stockholm, Sweden; 4 Universität Hamburg, CHyN, Luruper Chaussee 149, 22607 Hamburg, Germany; 5 Department of Chemical Engineering, Universitat Rovira i Virgili, Avinguda dels Països Catalans 26, 43007 Tarragona, Spain; 6 ICREA, Passeig Lluís Companys 23, 08010 Barcelona, Spain

## Abstract

In this manuscript, we demonstrate the applicability of a metabolic liquid biopsy for the monitoring and staging of patients with lung cancer. This method provides an unbiased detection strategy to establish a more precise correlation between CTC quantification and the actual burden of disease, therefore improving the accuracy of staging based on current imaging techniques. Also, by applying statistical analysis techniques and probabilistic models to the metabolic status and distribution of peripheral blood mononuclear cell (PBMC) populations “perturbed” by the presence of CTCs, a new category of adaptive metabolic pattern biomarker (AMPB) is described and unambiguously correlated to the different clinical stages of the patients. In fact, this strategy allows for classification of different categories of disease within a single stage (stage IV) before computed tomography (CT) and positron emission tomography (PET) scans and with lower uncertainty.

## Lung cancer: Blood test gauges metabolic differences to reveal disease stage

A new blood test could offer accurate staging information for patients with lung cancer. Ramon Alvarez-Puebla from ICREA and Universitat Rovira i Virgili in Tarragona, Spain, and colleagues developed the test using white blood cells seeded with lung cancer cells. The researchers introduced a fluorescent analog of glucose into the samples, which allowed them to identify tumor cells on the basis of elevated sugar consumption. The team evaluated this ‘metabolic liquid biopsy’ on blood samples from a small number of healthy donors and patients with metastatic lung cancer. The assay reliably differentiated between the two groups, with no CTCs detected in the healthy controls; patients with worse outcomes had higher CTC counts; and measuring the extent to which tumor cells affected the metabolism of non-cancerous cells in the same samples correlated with disease stage accounting for a new adaptative biomarker that allows for classification of different categories of disease.

## Introduction

Lung cancer is the second most common tumor malignancy and is a leading cause of cancer death worldwide in both, women and men (1.69 million deaths in 2015) http://www.who.int/mediacentre/factsheets/fs297/en/. According to American Cancer Society, the incidence of this disease was about 225,500 new diagnosed cases with 154,050 deaths in 2017 https://www.cancer.org/cancer/non-small-cell-lung-cancer/about/key-statistics.html. The mortality of lung cancer (non-small cell lung cancer, NSCLC; and small-cell lung cancer, SCLC) is very high, resulting from the early dissemination of cancer cells to secondary sites which are not detectable by conventional procedures.^1^ Notably, for stages I and IV, 5-year survival is 80 and 10%, respectively.^2^ Thus, early stages (I–II) are defined by the curability. However, even with radical surgery, between 30 and 50% of patients at early stages relapse.^3^ Stages III are even more heterogeneous, with a 5-year survival between 13 and 36%. Reason for these high-recurrence rates, even after radical surgery, is believed to be related to the presence of micrometastasis or lesions not detected by the diagnostic staging.^4^ Thus, methods that perform adequate staging are not only essential in the diagnosis, but also for detecting relapses or monitoring the response to therapy.

Currently, the lung cancer staging is based on (i) the tumor-node-metastasis (TNM) staging system, which typically relies on images acquired via computed tomography (CT) and positron emission tomography (PET); and (ii) the anatomopathological diagnosis.^5^ However, CT and PET scans suffer from major limitations in terms of resolution and accuracy. Among others, radiological lesions compatible with cancer are not always evident.^6^ Further, inflammatory or non-specific lesions can be erroneously identified as tumors in CT or PET.^7^ Mediastinal lesions (N2 or T4), which are critical factors in determining resectability of cancer, are characterized by an unsatisfactory diagnostic accuracy. In this regard, a meta-analysis including 45 studies has concluded that, with a standardized uptake value (SUV) cutoff of 2.5, the sensitivity and specificity of PET-CT were 77.4% and 90.1%, respectively.^8^

During the last years, precision medicine has undergone a great change in lung cancer treatment by describing very individualized new disease categories^9–11^ capable of effectively predicting the response to new treatments.^12^ However, the application of precision medicine requires the fine description of the patient disease at each specific state of the treatment, a task for which PET and CT scans often turn out to be insufficient. Thus, current clinical practice demands the definition of new biomarkers to resolve the limitations of CT and PET techniques. In this scenario, the quantification of circulating tumor cells (CTCs) in peripheral blood from cancer patients (liquid biopsy, LB) emerges as a non-invasive approach capable of overcoming the drawbacks associated with conventional imaging techniques.^13^ This new concept of biopsy relies on the assumption that CTCs detach from primary solid tumors, enter the bloodstream and, after extravasation, act as seeds for metastatic colonies.^14^ In this regard, it is worth noticing that not all tumor locations have the same behavior in terms of dissemination or invasion.^15^ Quantification of CTCs has shown to have independent prognostic values in colon, breast, and prostate cancer.^16^ Differently, studies of pancreatic cancer patients only displayed a general trend toward an association between CTC detection and disease progression.^17^ Although many approaches for CTC quantification have been developed in recent years, all of them are antigen-dependent, that is they rely on antibodies against epithelial receptors, such as the epithelial cell adhesion molecule (EpCAM).^18–20^ Therefore, these methods are unable to detect cells that do not express the pre-selected markers.^21^ Thus, current LB approaches “simply” enumerate a single subpopulation of CTCs, therefore failing to fully describe the whole heterogeneous ensemble of cancer cells, comprising epithelial tumor cells, cells that underwent the epithelial-mesenchymal transition (EMT), tumor stem cells, and even clusters of tumor cells.

Alternatively, instead of using markers targeting-specific membrane receptors,^22^ CTCs can be labeled by profiting from their characteristic metabolic activity (Warburg effect).^23^ In this approach, a glucose analogue labeled with a fluorophore is added to the cell sample containing CTCs. Then, the larger uptake of dye-labeled glucose molecules by tumor cells as compared with normal cells enables their detection by fluorescence spectroscopy.

In this manuscript, we demonstrate the applicability of this unbiased detection strategy to (i) establish a more precise correlation between CTC quantification and the actual burden of disease, therefore improving the accuracy of staging based on current imaging techniques (TNM), and (ii) recognize different categories of disease within a single stage (stage IV). First, we optimized the conditions for the selective labeling of CTCs, extracted from human blood samples through a partial enrichment method (Ficoll), for their identification and quantification independently of their phenotypic features. With this approach, all subpopulations of CTCs can be detected regardless of the histological characteristics of the primary tumor and the molecular variability associated with different stages of the EMT process. Second, by applying statistical analysis techniques and probabilistic models to the metabolic status and distribution of peripheral blood mononuclear cell (PBMC) populations “perturbed” by the presence of CTCs, a new category of adaptive metabolic pattern biomarker (AMPB) is described and unambiguously correlated to the different clinical stages of the patients.

## Results

**Fig. 1:**
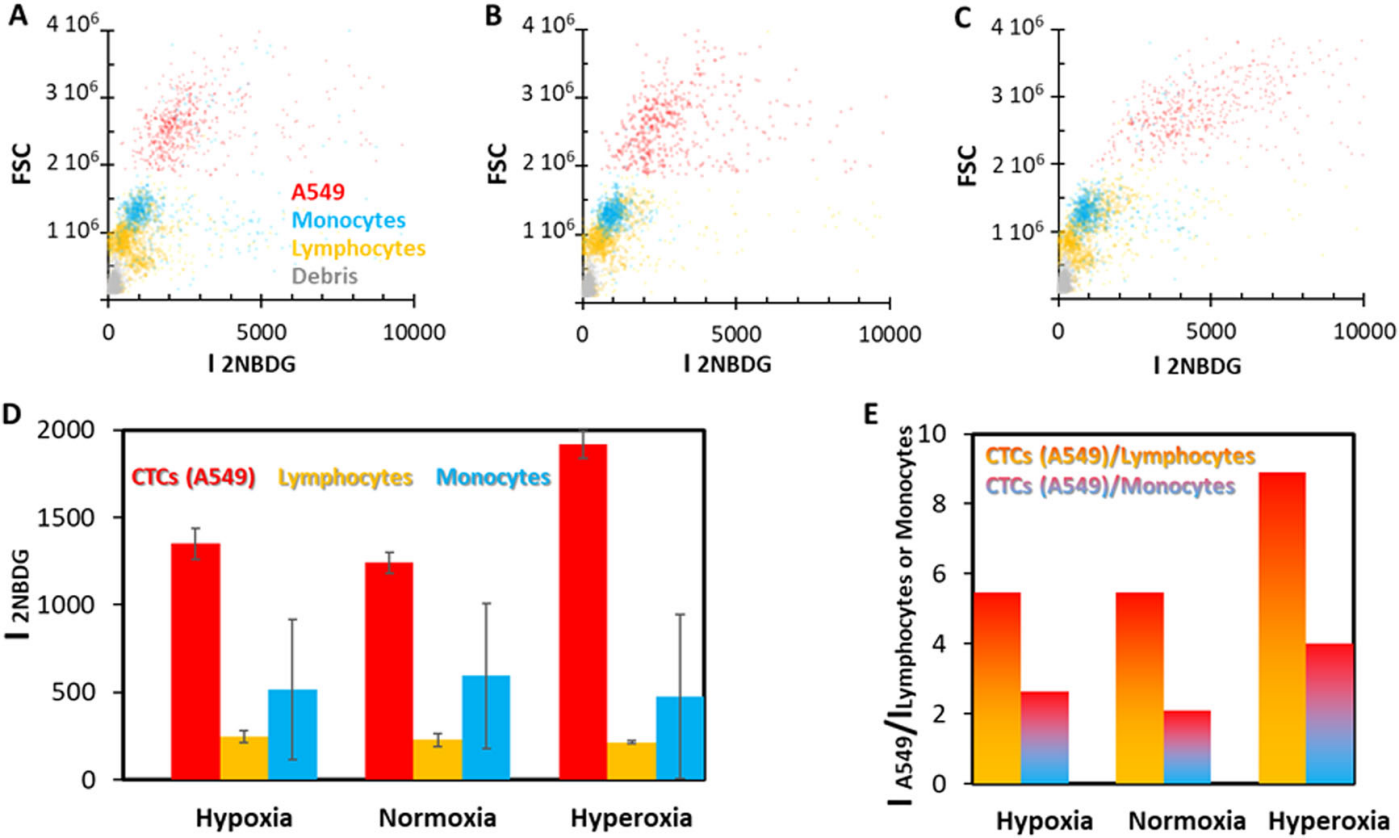
Effects of oxygen on human PBMCs spiked with a tumor cell line. Flow cytometry of PBMCs obtained from human blood from a healthy donor sample spiked with A549 cells (1:100 ratio, cancer:healthy) as a function of the oxygen conditions**. a** normoxia; **b** hypoxia; and **c** hyperoxia. **d** Average 2NBDG fluorescence emission per cell for the different oxygen conditions. **e** Ratiometric differences in 2NBDG fluorescence emission for cancer cells (A549) and PBMCs (lymphocytes and monocytes) under the three oxygen conditions. FSC: forward-scattered light

2-[N-(7-nitrobenz-2-oxa-1,3-diazol-4-yl)amino]-2-deoxy-d-glucose (2NBDG) is a non-toxic fluorophore characterized by a quantum yield of 0.55 and an absorption maximum at 465 nm, which generates an intense emission centered at 540 nm upon excitation with a blue laser line. Peripheral blood mononuclear cells (PBMCs) fractions (lymphocytes and monocytes), extracted through a Ficoll process from human blood samples obtained from healthy donors, were spiked with a lung cancer cell line (adenocarcinoma human alveolar basal epithelial cells, A549) in a 1:100 ratio (cancer:healthy), and later incubated with a cocktail of: (i) 2NBDG, (ii) anti-leukocyte common antigen (CD45) labeled with allophycocyanin (CD45-APC, absorption: 650 nm, emission: 660 nm), and (iii) anti-cluster of differentiation 14 (CD14) labeled with peridinin-chlorophyll/Cyanine5.5 (CD14-PerCP/Cy5.5; absorption: 482 nm, emission: 676 nm). Figure 1a shows the cytometry results for this sample measured under optimized conditions: 300 µM concentration of 2NBDG, 30 min of incubation time, and normoxia. The cytogram exhibits three populations. Specifically, CD45+/CD14−/2NBDG−, CD45+/CD14+/2NBDG+, and CD45−/CD14−/2NBDG+ events are ascribed to lymphocytes, monocytes (macrophages and dendritic cells), and cancer cells (A549), respectively. Gray events, negative for all labels, are considered debris.

Normal PBMCs and tumor cells display clear differences in 2NBDG fluorescence intensity, which are consistent with their different metabolic activity. As previously demonstrated,^23^ and in full agreement with the Warburg effect, the 2NBDG uptake can be further increased by adjusting the oxygen concentration. In this regard, while the oxygen content has no remarkable effect on the 2NBDG fluorescence of PBMCs, both hypoxia (Fig. 1b) and hyperoxia (Fig. 1c) conditions yield larger fluorescence signals for A549, being significantly larger in the latter case (Fig. 1d). On the other hand, ratiometric 2NBDG fluorescence emission measurements for cancer cells vs. PBMCs (Fig. 1e) clearly show that hyperoxia conditions (300 μM concentration of 2NBDG, 30 min incubation time) are the best suited to maximize the fluorescence ratios (i.e., higher discrimination capabilities). It is worth noting that longer incubation times and/or larger 2NBDG concentrations are avoided as they promote apoptosis or autophagy due to the depletion of growth factors (Figure S1).

**Fig. 2:**
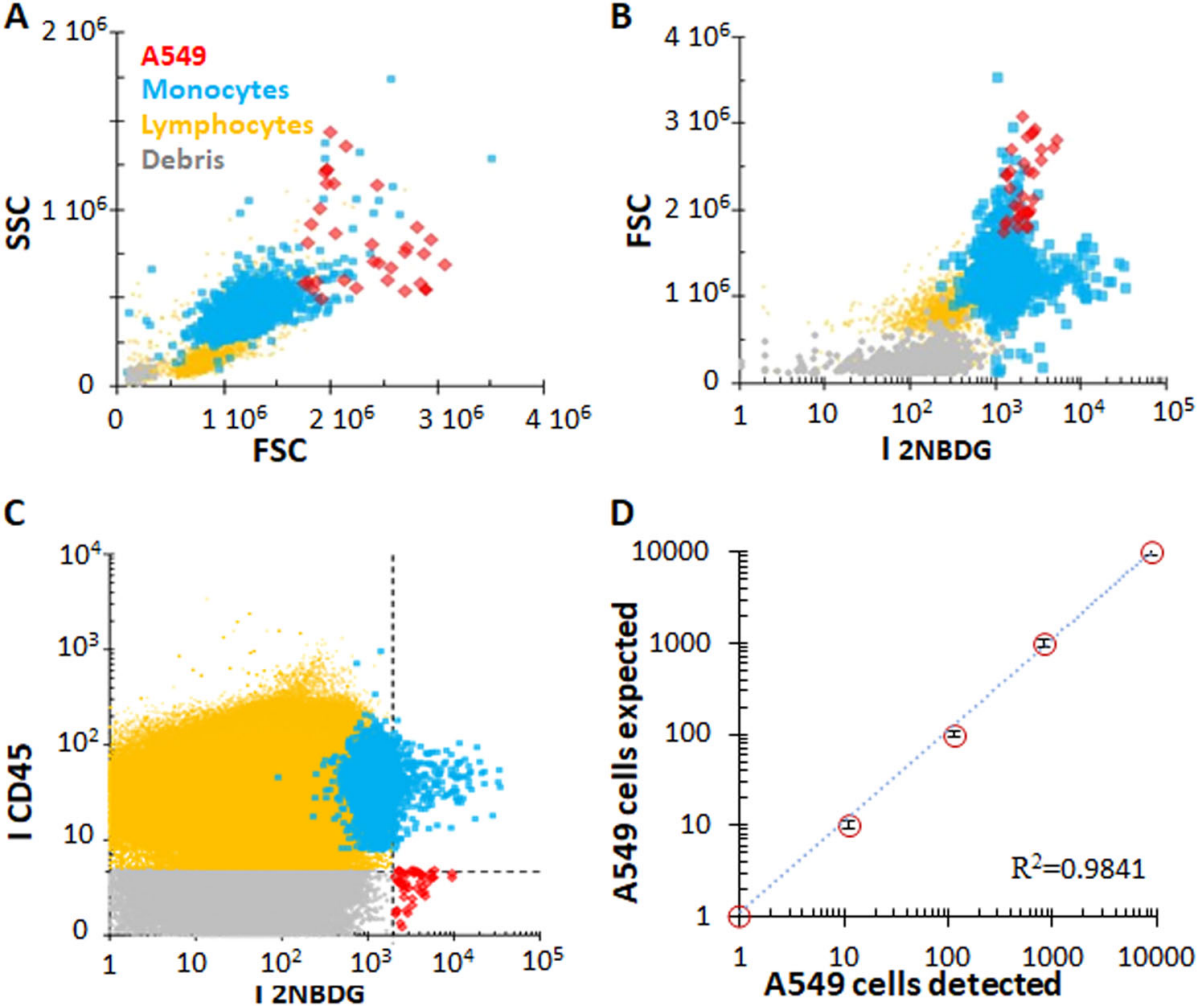
Gating strategy and correlation between detected and expected CTCs in human PBMCs. Flow cytometry distribution of cells PBMC sample obtained from human blood from a healthy donor sample spiked with A549 cells (1:10^4^ ratio, cancer:healthy) as a function of the **a**. cell complexity and size; **b** 2NBDG fluorescence emission and size; and **c** 2NBDG and CD45 fluorescence emissions. **d** Correlation between detected and expected A549 cells per million of cells for different cancer:healthy cells ratios (1:10^2^, 1:10^3^, 1:10^4^, 1:10^5^, and 1:10^6^). SSC: side-scattered light

The detection limits of the method were tested by analyzing samples containing progressively diluted tumor cells spiked into PBMCs suspensions, from 1:10^2^ to 1:10^5^ A549:PBMC cell ratios. These suspensions were then incubated with 2NBDG, CD45-APC, and CD14-PerCP/Cy5.5, under hyperoxia conditions. As in the previous cases (Fig. 1a–c), the cytograms show four populations composed by CTCs, lymphocytes, monocytes, and debris (Fig. 2a, 1:10^4^ ratio). However, the representation of the 2NBDG fluorescence intensity vs. the forward scattering (Fig. 2b) reveals that, for samples containing a small fraction of CTCs, the differentiation between A549 and monocytes is no longer accomplished based solely on the 2NBDG marker. This is likely because some of these PBMC cells may consume large quantities of glucose, thus generating false positives.^23^ Such issue is overcome by integrating the CD45 signal as an additional discrimination parameter. In fact, by representing the fluorescence intensity of 2NBDG against that of CD45 (Fig. 2c), a panel is generated were the different populations can be unambiguously classified into four different quadrants: up-left (CD45+/2NBDG−), lymphocytes and monocytes; up-right (CD45+/2NBDG+), activated monocytes; down-left (CD45−/2NBDG−) debris; and down-right (CD45−/2NBDG+), CTCs. Figure 2d shows the representation of the number of A549 cells spiked into the PBMC sample vs. the number of expected CTCs at different cell ratios, revealing a remarkable degree of correlation (*R*^2^ = 0.9841). Outstandingly, these data show that, for all samples, cells with a positive 2NBDG response and a lack of CD45 signal (i.e., CTCs) display a larger 2NBDG fluorescence emission as compared to normal cells, which is sufficient to detect single recognition events even at highly diluted regimes.

**Fig. 3:**
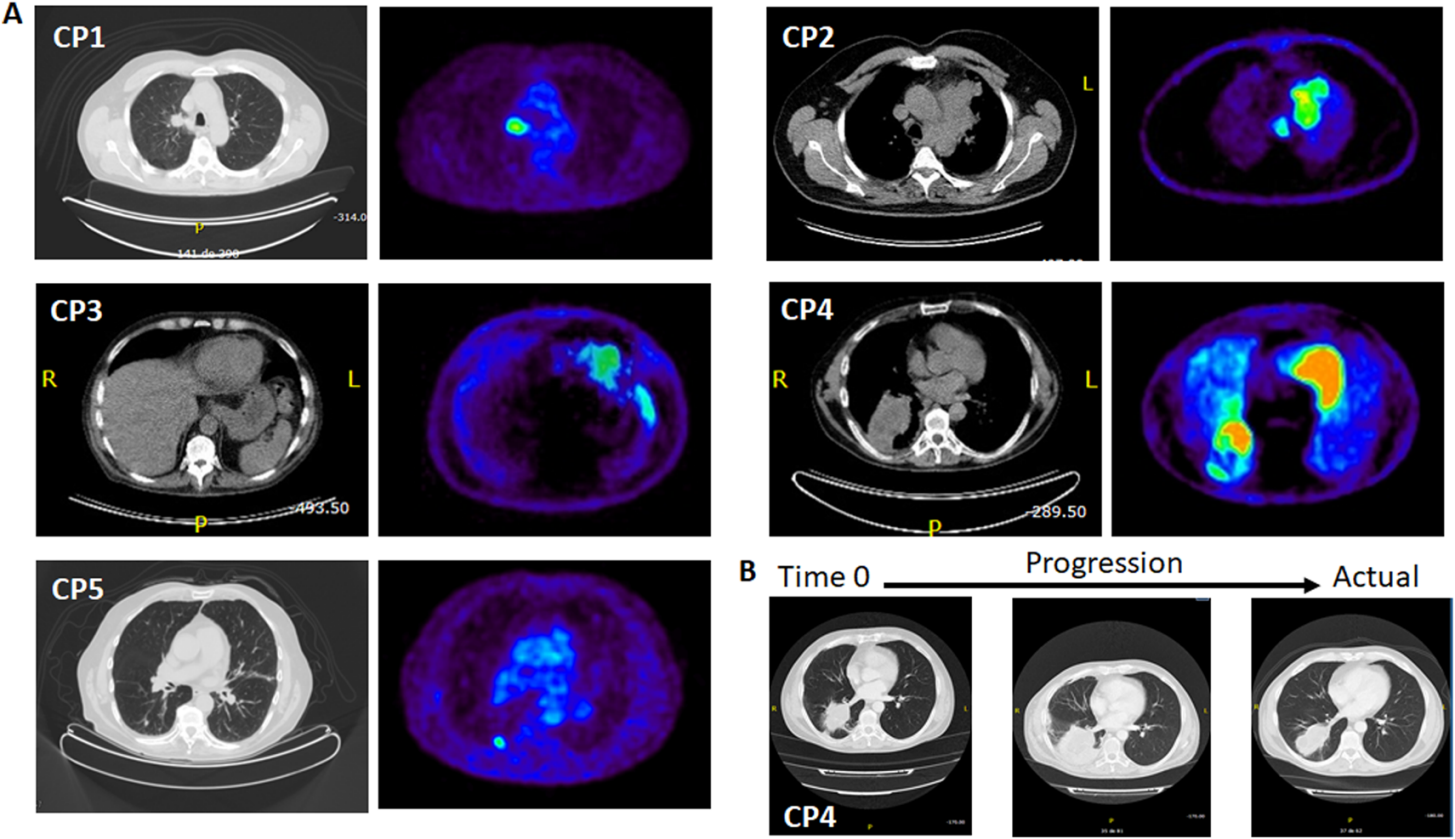
Computed tomography Positron emission tomography scanners. **a** Cross-sectional CT and PET-CT scans corresponding to the maximum size of the primary tumor from five patients. All patients are in stage IV (metastatic) but with different extensions and size of the primary tumor. CP1, adenocarcinoma patient with a non-bulky primary tumor, but with a very wide metastatic extension (subcutaneous); CP2 and CP3, CA patients with large primary tumors (bulky) but with limited extensions of metastasis; CP4, a priori, patient with a large primary tumor (bulky) with limited extension of metastasis (**b**); CP5, patient with a non-bulky primary tumor with limited extension of metastasis. **b** CT scan cross-sections showing the tumor progression of CP4 with treatment

**Fig. 4:**
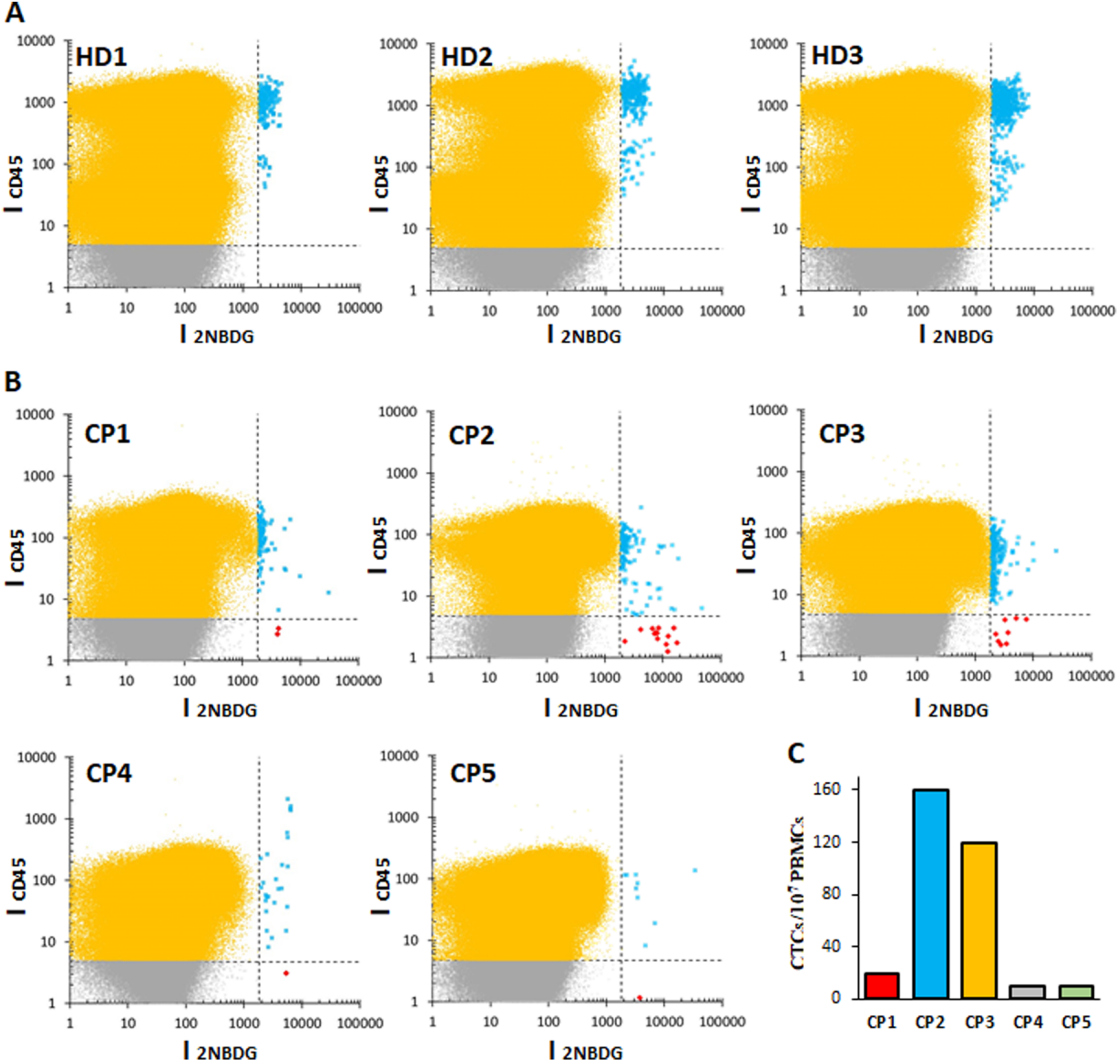
Flow cytometry data of samples of healthy donors and patients. **a** Healthy donors and **b** Lung cancer patients, as a function of 2NBDG and CD45 fluorescence emissions. **c** Number of CTCs per 10^7^ PBMCs

Proof of concept clinical application of this method was demonstrated by quantifying CTCs in real blood samples obtained from different healthy donors (three) and selected lung cancer patients (five). All patients were diagnosed with metastatic lung cancer, but with different histology and disease distribution. Their clinical characteristics at the time of the blood extraction are summarized in Table 1, while Fig. 3a shows the correspondent disease extension through CT and PET scans. Samples (8 mL) from healthy donors and patients were treated through density gradient (Ficoll) to extract the peripheral blood mononuclear cells, and subsequently stained with CD45 and 2NBDG, before running them into the flow cytometer. Figure 4 shows the results obtained as a function of the fluorescence intensities of 2NBDG vs. CD45. As in the case of spiked samples, positive CTC recognition events were defined for a CD45−/2NBDG+ response. As expected, healthy donors show no evidence of CTCs while patients display, for all the cases, a variable number of tumor cells. These data correlate well with those obtained with conventional diagnosis at the time of sample extraction (Tables 1 and 1S, and Fig. 3a). First, patients at partial response (CP1) or complete remission (CP5) exhibit a very low number of CTCs (20 and 10 CTCs per 10^7^ PBMCs, respectively). Conversely, those patients with bulky disease (CP2 and CP3) show a considerably larger number of CTCs (160 and 120 CTCs per 10^7^ PBMCs, respectively). Notably, for CP4 some disagreement appears between the PET scan (Fig. 3a) and its high SUV, and the low number of CTCs (10 CTCs per 10^7^ PBMCs). However, CP4 represents a clear example of the paradoxical radiological response of some patients when treated with immunological drugs (i.e., pseudoprogression). In these cases, although, the patient’s clinical situation may appear as a progression of the disease, in reality, the tumor is in remission as clearly demonstrated by CT monitoring over the time after several treatment cycles (Fig. 3b).^24^

**Table 1:** Clinical characteristics and tumor extension at the time of blood extraction

	Anatomopathological diagnostic	UICC stage	PET SUV	Primary tumor	Metastasis localization	CTCs/10^7^ PBMCs
CP1	Adenocarcinoma	IV	7.44	Multi-metastatic^a^ Non-bulky	Lung Mediastinal Subcutaneous Skin	20
CP2	Small-cell lung cancer	IV	15.5	Pauci-metastatic^b^ Bulky	Mediastinal Lung	160
CP3	Undifferentiated CA	IV	6.23	Pauci-metastatic Bulky	Mediastinal Lung	120
CP4	Epidermoid CA	IV	19.8	Pauci-metastatic Bulky (false)	Mediastinal Lung Adrenal	10
CP5	Adenocarcinoma	IV	6.41	Pauci-metastatic Non-bulky	Mediastinal Lung	10

*UICC* Union for International Cancer Control, *SUV* standardized uptake value, *CTC* circulating tumor cell, *PBMC* peripheral blood mononuclear cell, *CA* carcinoma

^a^Multi-metastatic, more than three metastatic localizations

^b^Pauci-metastatic, three or lower metastatic localizations

**Fig. 5:**
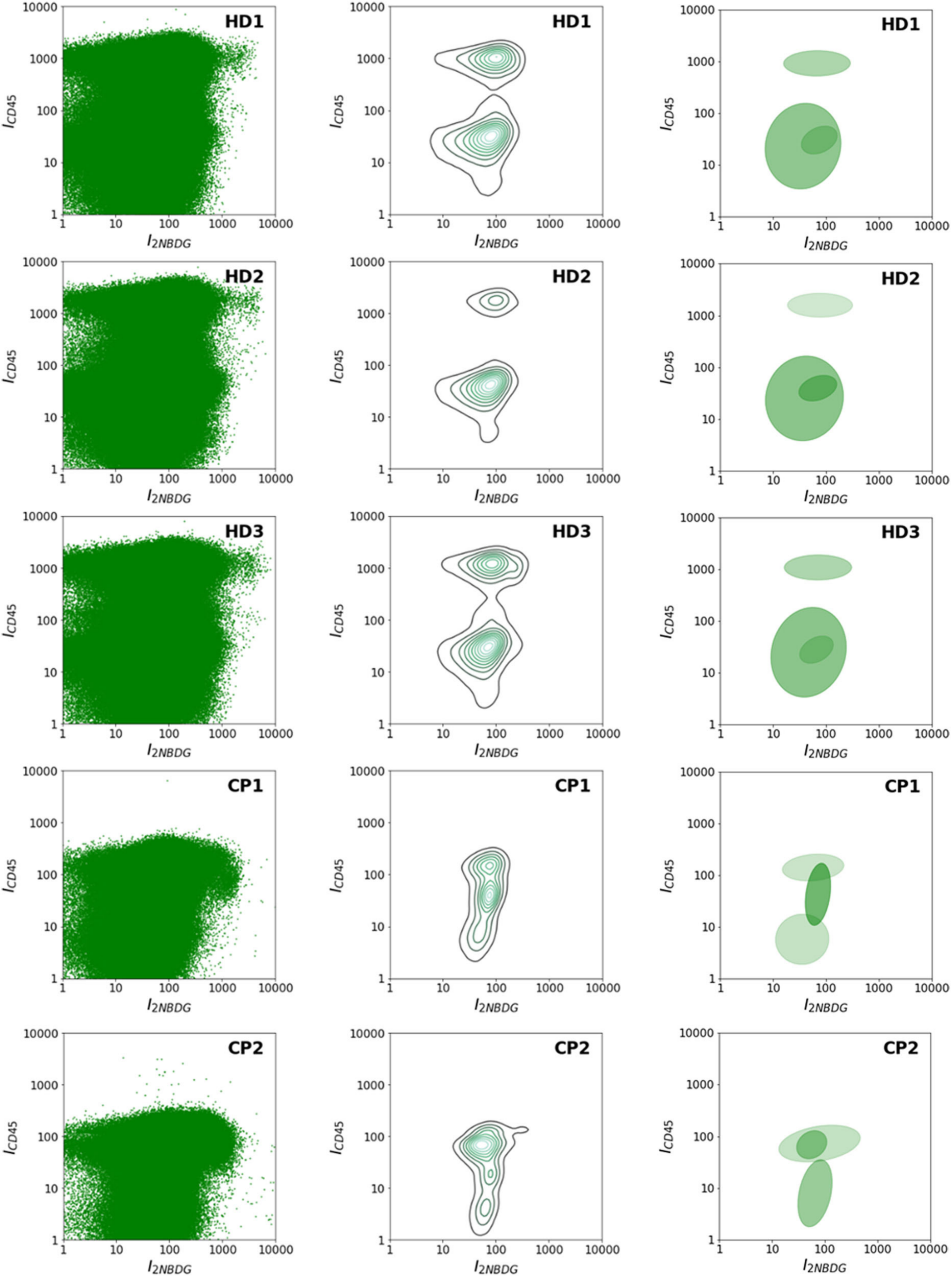

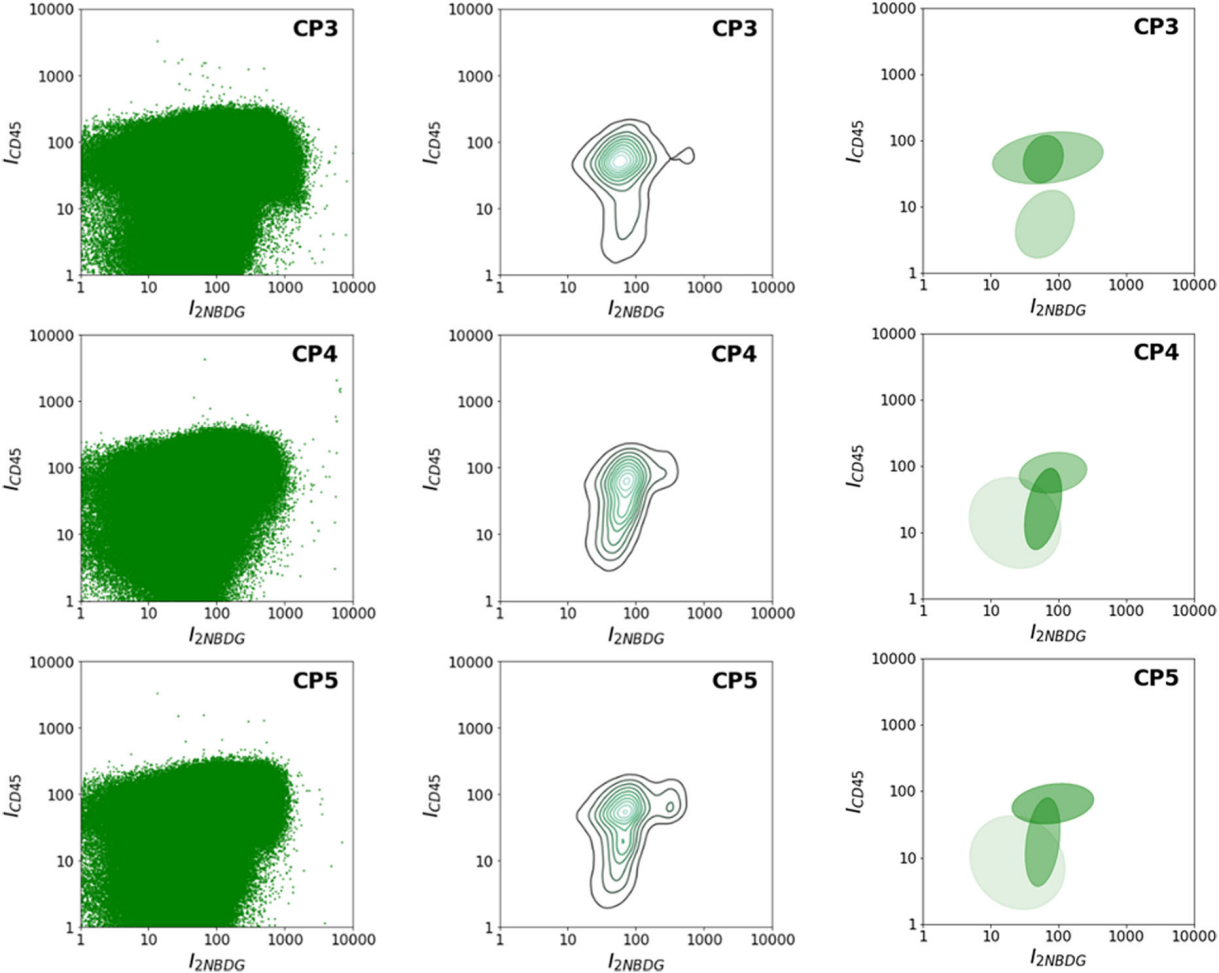
**a** Uncategorized, kernel density, and Gaussian mixture models of the flow cytometry row data. Uncategorized flow cytometry raw data, as a function of 2NBDG and CD45 fluorescence emissions; **b** kernel density estimate of the distribution and **c** Gaussian mixture models, of PBMC samples obtained from healthy donors (HD) and lung cancer patients (CP)

**Fig. 6:**
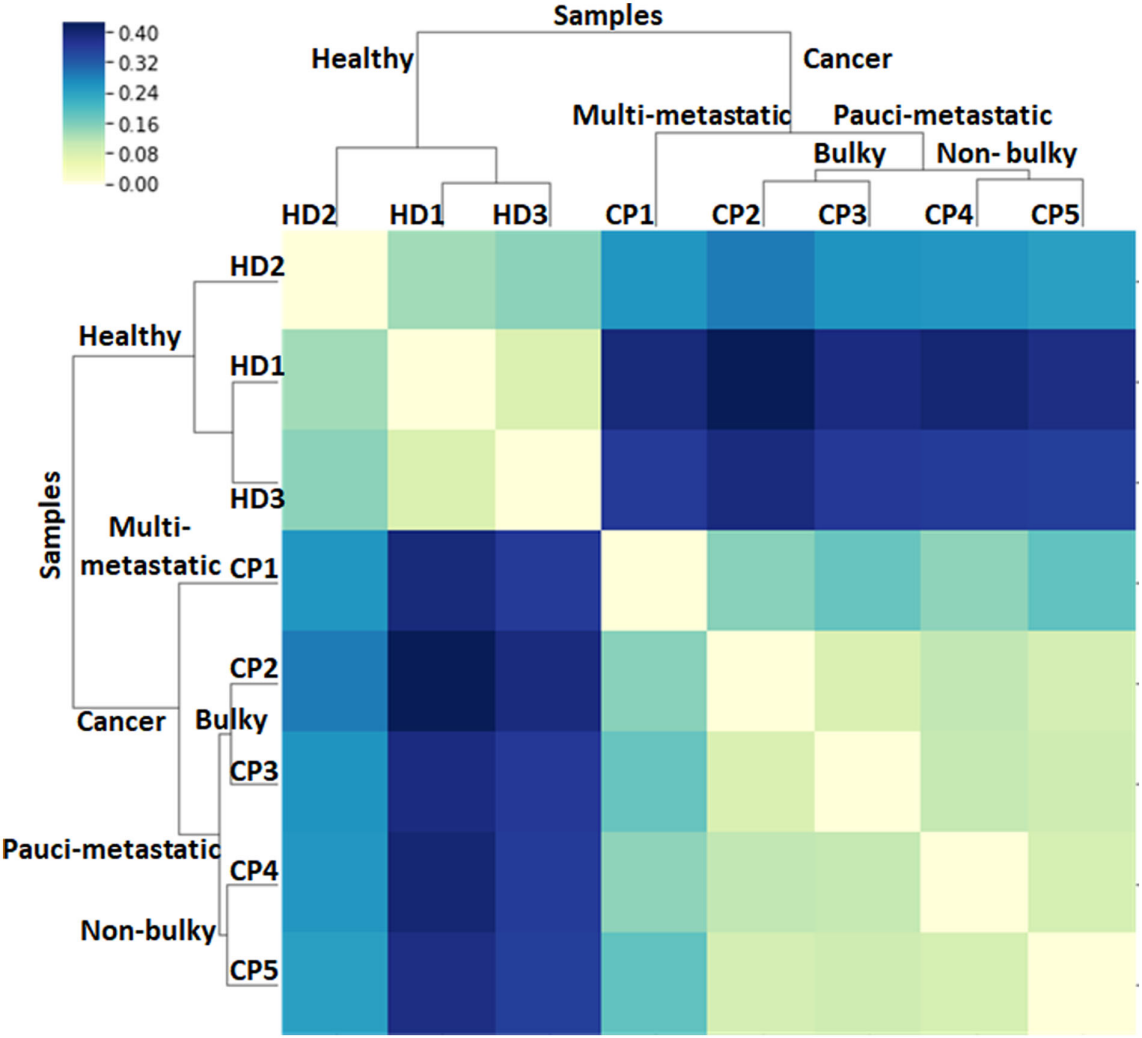
Two-dimensional KS statistic and unsupervised hierarchical clustering of healthy donors and cancer patients. From the distribution of the raw data, distances between all pairs of samples were calculated using the two-dimensional KS statistic.^26^ Then individuals where grouped using hierarchical clustering

Interestingly, the cytometric panels in Fig. 4 reveal different patterns of PBMC population distribution between healthy donors and cancer patients, which can be clearly discerned even by the naked eye. For instance, it is evident that the PBMC population presents a more compact distribution in patients as compared to healthy donors. To check the potential information hidden in these patterns, several statistical, and probabilistic data analysis methods were applied. As a first approach, all raw data, including debris, PBMCs, and CTCs (Fig. 5a), were profiled by using kernel density estimates of their distribution (Fig. 5b).^25^ In these distributions, healthy individuals show two cell populations (corresponding to the two peaks of the distribution) as compared to the single, broader cell cluster exhibited by cancer patients. Motivated by the observation of distinct cell populations, especially in the case of healthy individuals, Gaussian mixtures were used to model each distribution (Fig. 5c).^25^ These models uncover several characteristic fingerprints. First, all healthy donors present a cell population at high-CD45 intensities (over 1000). Second, two other populations are clustered at intensities in the 10–100 range for both 2NBDG and CD45. In contrast, the high-CD45 intensity population is absent for all patients, while the single collection at lower CD45 intensity shown in Fig. 5b results in three subgroups that are characteristic of the patient diagnostic/prognostic. Notably, according to these graphs, the cancer patients can be subcategorized in three different groups: CP1, CP2-3, and CP4-5. Independently, the distributions of the raw data were compared by using the two-dimensional Kolmogórov-Smirnov (KS) statistic^26,27^ to acquire an unsupervised clustering of individuals (Fig. 6). In particular, the distance between pairs of distributions was computed. Then, hierarchical clustering was applied to group the individuals. Again, healthy individuals are clearly separated from patients, while patients are classified in the same three subgroups as before: CP1, CP2-3, and CP4-5.

Notably, this categorization in three subgroups only partially agrees with that arising from CTC quantification (i.e., CP1, CP4-5: low-CTC content, vs. CP2-3: high-CTC content; Table 1). Notwithstanding, these different interpretations of the cytograms may reveal two complementary dimensions of the disease. The first one, CTC enumeration, takes account of the disease burden of the primary tumor, while the other, the analysis of the cell population pattern, appears to correlate well with the disease extension. This latter observation is clearly corroborated by the results of the CT and PET scan images and the volume of the primary tumor (Fig. 3a). In this regard, by applying conventional radiological criteria, the disease can be defined as multi-metastatic (extensive) or pauci-metastatic (limited), while the volume of the primary tumor can be differentiated as bulky or non-bulky. Following these conventionalisms, CP1 presents a more extended disease (multi-metastatic, lung, mediastinal, subcutaneous, and skin) with no dominant localization, not even in the primary tumor (non-bulky). The rest of the patients (CP2-5), however, display a limited disease (pauci-metastatic), but with different volumes in the primary tumor, bulky for CP2-3 (active disease) and non-bulky for CP5 (in remission). In the case of CP4, again, the monitoring of the disease with imaging techniques (Fig. 3b) revealed that the initial bulky disease shown in the CT and PET scans (Fig. 3a) was paradoxical, which could be ascribed to the immunotherapy (the clinical evolution showed that a considerable part of the supposed tumor mass was due inflammation and infection, Fig. 3b).

## Discussion

Early and accurate diagnosis of lung metastasis is a pressing clinical need. Lung cancer is prevalent and, in many cases, difficult to detect or to stage with accuracy by conventional imaging. Failing to accurately establish the existing stage of the disease severely impacts the quality of diagnosis, as the actual threshold guides the therapeutic strategy from potentially curative to palliative. Important limitations of the imaging techniques, such as at determining the presence of lymph nodes and mediastinal metastasis or discriminating real tumor from reactive processes, makes the development of new diagnostic biomarkers necessary. This could also be useful to evaluate the responses to treatment and, once validated, even be helpful for early diagnosis.

To date, the main goal of conventional LBs is identifying the relationship between the number of CTCs and the patient prognostics by either measuring progression-free survival (PFS) or overall survival (OS).^28^ In this way, it had been possible to determine thresholds for the CTC content from which prognostics is clearly differentiated (at least for colon, breast and prostate cancer).^16^ Conversely, the correlation between the clinically defined tumor burden and extension, and the number of CTCs has never been established. Further, available LBs^29^ intrinsically underestimate the number of CTCs in blood due to their antigen-based approach, which limits the count of positive events to the sole recognition of cells with epithelial receptors in their membrane (i.e., EpCAM),^22^ while mesenchymal, tumor stem cells or fluctuating phenotypic CTCs^30,31^ are completely disregarded. This represents a major issue. For example, a recent study conducted in patients with lung cancer undergoing radical surgery (early stages) showed that the highest preoperative clinical staging was related to the increased presence of mesenchymal CTCs, but not with epithelial cells.^32^ In addition, a comparative enumeration of CTCs of the same sample obtained by using EpCAM with CellSearch® (the only FDA approved LB) and cytometry showed that the former technique only accounts for a third of CTCs events yielded by cytometry.^33^ Thus, to date, it is unknown whether the prognostic value revealed by these CTC quantification studies reflects the radiologic volume/stage of the disease or is correlated with other biological characteristics, such as those associated with aggressiveness, invasiveness, or the type of CTC detected.^34,35^

Here we have developed a metabolic approach to the liquid biopsy that can sensitively, specifically, and safely detect the presence of CTCs in model patients with lung cancer at stage IV. This metabolic liquid biopsy (MLB) radically redefines the sensing strategy, profiting from the different consumption of glucose by tumor cells as compared with healthy ones (Warburg effect).^23^ With this approach, two complementary layers of information are provided. First, by using a metabolic feature universally shared by all tumor cells,^36^ MLB has the capability of discriminating and quantifying all CTCs (mesenchymal and epithelial), rather than only the epithelial subgroup as for conventional phenotypical LBs. Second, as MLB also labels all PMBCs, it is possible to extract metabolic patterns using appropriate statistical and probabilistic analysis which informs about the impact of the CTC presence on the rest of the non-cancer cells. Thus, by applying two independent statistical approaches to the cytometric data, another dimension of the disease is profiled by revealing a new class of adaptive metabolic pattern biomarker (AMPB), which describes the extension of the disease as a function of the number of metastasis. Within our cohort, we selected patients with lung cancer at the same disease stage but with different disease distribution (defined radiologically with CT and PET scans). The selection was made based on (i) the presence or not of a bulky tumor (massive primary tumor), and (ii) the extent of the metastasis. This last feature was defined as the presence of more or <3 metastatic sites (multi- vs. pauci-metastatic disease) that would correspond to the stages M1a/M1b. Based on the CTC enumeration, patients were discriminated into those with high (CP2-3) and low (CP1, CP4-5) load of tumor cells. Subsequently, the AMPB extracted from MLB clearly defined, for patients at the same clinical stage, the presence or absence of a primary tumor mass (bulky); a condition which is mostly related to the overall number of CTCs. It is worth reminding that CP4 is the patient who presented a paradoxical response in the PET because of immunotherapy and inflammation.

While described for diagnosis and stratification in patients with radiologically stage IV lung cancer, we believe that this MLB approach may be also amenable to the detection of true stages IV when the radiological diagnosis inaccurately establishes a stage I or II (30–40% of relapses after radical surgery). In these situations, unnecessary surgery could be avoided. On the other hand, in stages III, where a very heterogeneous prognosis occurs (from 13 to 36% of 5-years survival rate), MLB would allow rescuing those patients susceptible of curative surgery. Joining a list of recent next-generation diagnostics (circulating tumor cell assays, biomarker monitoring), MLB-coupled to big data analysis (adaptive metabolic pattern biomarker, AMPB) aims to build toward early identification, prognosis, and staging of metastatic disease that may result in improved patient outcomes and decreased treatment toxicities. With a growing population of patients at risk of developing lung metastasis, a highly sensitive, non-surgical, nonradioactive method for repeated monitoring may be clinically useful. Although these preliminary data show solid evidence on the effectiveness of the method with the minimum human cost validation of our findings with prospective clinical trials testing therapeutic strategies based on MLB assessment will be required to establish clinical utility.

## Methods

### Study design and patients

Patients and healthy adult blood donors were recruited through the Grupo HM Hospitales. Personal and clinical data were recorded according to standard clinical procedures. All samples were collected with written informed consent and institutional review board (Ethics and Clinical Research Committee of the Grupo HM Hospitales. Madrid, Spain; www.hmhospitales.com) approval in accordance with the Declaration of Helsinki. The set of patients selected for this study was identified from patients diagnosed and treated for stage IV of lung cancer. Five of them were recruited with the primary goal of analyzing the association of the number of CTCs and the metabolic distribution of PBMCs with the diagnostic of lung cancer. Eligible patients included in this study were age ≥18 years with treated primary lung cancers at stage IV with either non-small cell lung cancer (NSCLC) (adenocarcinoma and squamous cell carcinoma) or small-cell lung cancer (SCLC) histology and receiving treatment with chemotherapy or immunotherapy. Eligible patients underwent pretreatment imaging by chest CT and whole-body PET-CT. All patients underwent pretreatment brain MRI. Patients were chosen based on the volume of their primary tumor, considering two categories: (i) tumor size (bulky, >4 cm, and non-bulky, <4 cm); and (ii) metastasis extension (extended or multi-metastatic, >3 M1a/M1b, and not-extended or pauci-metastatic, <3 M1b).

## Experimental

### Cell culture

A549 lung cancer cells were obtained from the American Tissue Culture Collection (ATCC, Manassas, VA, USA) and cultured in RPMI 1640 media supplemented with 10% fetal bovine serum. Cells were maintained at 37 °C in a humidified 5% CO_2_ atmosphere.

### Clinical samples

Personal and clinical data were recorded according to standard clinical procedures. All specimens were obtained with informed consent. The study was approved by the local Ethics and Clinical Research Committee. A volume of 8 mL of blood was drawn in 10 mL vacutainer tubes containing ethylenediaminetetraacetic acid (EDTA) and processed in the first 24 h. Results for CTCs were linked to clinical data.

### Preparation of samples

Blood samples from cancer and healthy patients were obtained from the Servicio de Oncología of the HM Hospital Universitario Torrelodones-Madrid. Peripheral blood mononuclear cells (PBMCs) were isolated from the whole blood using Ficoll-Paque PLUS (purchased from GE Healthcare Life Science). A volume of 15 mL of Ficoll solution was added to a 50 mL Leucosep centrifuge tubes (Greiner Bio One) and blood was disposed as a layer onto Ficoll. Samples were centrifuged at 800 g for 15 min at 18 °C, and the resulting PBMC layer was separated from the rest of phases. PMBCs were washed twice in 10 mL phosphate buffered saline (PBS) by centrifugation at 250×*g* for 10 min and finally suspended in RPMI 1640 supplemented with 10% FBS, until use. Hypoxia and hyperoxia conditions were achieved by placing a PBS solution of 2NBDG in a closed tube (15 mL sterile disposable centrifuge tube, Fisherbrand) inside modular incubator chambers (Billups-Rothenberg, Inc). Air-tight chambers were connected to a nitrogen gas line to replace all oxygen content (hypoxia conditions) or connected to an oxygen line to provide an extra amount of oxygen saturation (hyperoxia conditions) during 20 min. Levels of oxygen saturation were measured to determine the quantity of oxygen in the system even after 1 h.

### Cell line uptake studies

A certain amount of A549 (ATCC® CCL185™), or MCF7 (ATCC® HTB-22™), cancer cells were spiked into collected PBMCs from healthy donors. Cells were pelleted and placed in 100 µL PBS supplemented with 10% FBS. A volume of 4 µL anti-CD45-APC (for leukocytes staining) or anti-CD14-PerCP/Cy5.5 (for monocytes), were added. Additionally, 100 µL of 2NBDG at different concentrations was added: 50 µM, 300 µM, and 600 µM; in time, 10 min, 30 min and 60 min. Afterwards, cells were washed twice by centrifugation at 250×*g* for 10 min and suspended in cold PBS. The same procedure was followed to study the oxygen availability (normoxia, hyperoxia, or hypoxia) effect on the uptake of 2NBDG by cells, choosing 300 µM 2NBDG and 30 min as optimal conditions. Moreover, different amounts of A549 cancer cells were selected and spiked into the blood: 10,000; 1000; 100 and 10 cells for 1 × 10^6^ PBMCs. The same amounts of stains were added and 300 µM and 30 min of time under hypoxia conditions were used for the 2NBDG incubation.

### Healthy and cancer patient blood uptake studies

PBMCs from cancer patients enrolled in the study were directly pelleted and placed in 100 µL PBS supplemented with 10% FBS. A volume of 100 µL of 2NBDG of 300 µM and 4 µL of anti-CD45-APC (for leukocyte staining), were added for 30 min, under hyperoxia at room temperature (RT). Afterwards, cells were washed twice by centrifugation at 250×*g* for 10 min and suspended in cold PBS.

### Sample measurements with flow-cytometry

Flow cytometry of the samples was carried out in a NovoCyte Flow Cytometer (from AceaBiosciences), equipped with 488 nm and 640 nm excitation lasers and 530/30 nm and 675/30 nm detection filters. The 488 nm blue laser and 530/30 nm filter were used for the excitation and collection of 2NBDG staining, The 675/30 nm filter was used to collect fluorescence from PerCP/Cy5.5 excited with the same laser. The 640 nm red laser and 675/30 nm filter were used for excitation and emission collection from APC dye. Cytometry data were analyzed with NovoExpress and FloJo VX software and Microsoft Excel.

### Statistical analysis and probabilistic modeling

The kernel density estimates and the Gaussian mixture models were obtained using the Scikit-learn Python module.^25^ The Gaussian mixture models were fitted (including full covariances) using variational inference with a fixed number of components (*n* = 3) and Dirichlet priors. All other parameters and hyperparameters were set to their default values in Scikit-learn. The two-dimensional KS statistic was calculated using our own implementation of the algorithm described in ref.^26^ based on the implementation in ref.^27^ Because calculation of the two-dimensional KS statistic is computationally demanding, we used 50% of each dataset for all pairwise comparisons.

### Data availability

The authors declare that the data supporting our findings are included in the paper. Raw data are available upon reasonable request to the authors.

## Electronic supplementary material

Supplementary Information

## Electronic supplementary material

**Supplementary information** accompanies the paper on the *npj Precision Oncology* website (10.1038/s41698-018-0059-9).
