# Supplementary material for: Adaptive metabolic pattern biomarker for disease monitoring and staging of lung cancer with liquid biopsy
Source: NPJ Precis Oncol. 2018 Aug 8;2:16. doi: 10.1038/s41698-018-0059-9 (PMC6082903; doi:10.1038/s41698-018-0059-9)
Supplement: Supplementary file 1 — Supplementary Information [file 41698_2018_59_MOESM1_ESM.pdf]

Supplementary Information

Adaptive Metabolic Pattern Biomarker for disease monitoring and staging of lung cancer with liquid biopsy

Manuel Garcia-Algar,[1] Ana Fernandez-Carrascal,[1] Ana Olano-Daza,[2] Luca Guerrini,[1] Neus Feliu,[3,4] Wolfgang J. Parak,[4] Roger Guimera,[5,6] Eduardo Garcia-Rico,[2]* and Ramon A. Alvarez-Puebla[1,6]**

[1] Department of Physical Chemistry, Universitat Rovira i Virgili, Marcel•lí Domingo 1, 43007 Tarragona, Spain.

[2] Department of Medical Oncology, Hospital Universitario HM Torrelodones, Castillo de Olivares s/n, 28250 Torrelodones (Madrid), Spain.

[3] Karolinska Institutet, Stockholm, Sweden.

[4] Universität Hamburg, CHyN, Luruper Chaussee 149, 22607 Hamburg, Germany.

[5] Department of Chemical Engineering, Universitat Rovira i Virgili, Avinguda dels Països Catalans 26, 43007 Tarragona, Spain.

[6] ICREA, Passeig Lluís Companys 23, 08010 Barcelona, Spain.

Supplementary Table S1. Personal and clinical characteristics of the patients.

	CP1	CP2	CP3	CP4	CP5
**Age**	65	56	65	59	71
**ECOG [1]**	0	0	0	0	0
**Gender**	Male	Male	Male	Male	Male
**Stage at analysis**	IV	IV	IV	IV	IV
**Previous surgery**	No	No	No	No	No
**Radiotherapy**	Yes	Yes	No	No	Yes
**Chemotherapy**	CDD [2]	CDD	CDD	CDD/ immunotherapy	CDD
**Time from last chemotherapy**	15 days	6 days	15 days	14 days	30 days
**Pathologic diagnostic method**	Bronchoscopy	Bronchoscopy (Node puncture)	Transthoracic (Core needle biopsy)	Bronchoscopy	Transthoracic (Core needle biopsy)
**Molecular biomarkers**		No (SCLC)	No (Undifferentiated CA)	No (Epidermoid CA)	
- **EGFR [3]**	Not mutated,				Not mutated,
- **EML4[4]/ALK[5]**	Not translocated				Not translocated
- **ROS1[6]**	Not rearrangement				Not rearrangement

[1] Eastern Cooperative Oncology Group (Scale of Performance Status); [2] cis-diamminedichloroplatinum; [3] Epidermal growth factor receptor; [4] Echinoderm microtubule-associated protein-like 4; [5] Anaplastic lymphoma kinase; [6] Proto-oncogene tyrosine-protein kinase ROS1 gene

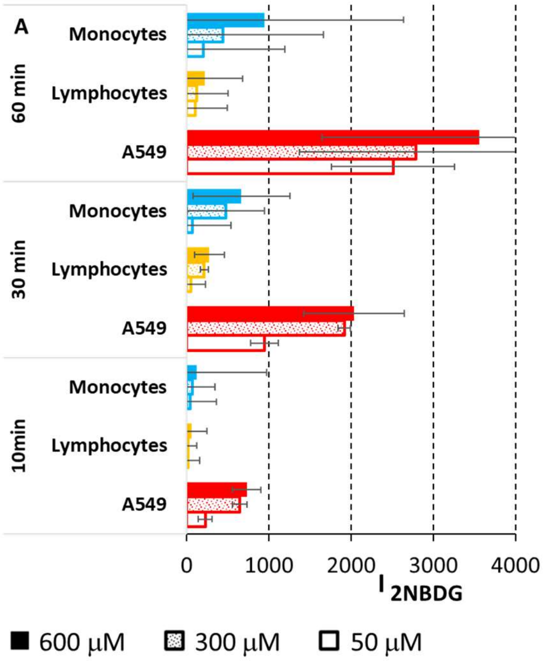

Supplementary Figure S1. Comparison of the degree of metabolic labeling for two different tumor cell lines (A549 and MCF7) diluted in human PBMC (1:100 ratio, cancer:healthy) as a function of time and concentration of 2NBDG.
